# Overcoming Resistance to Immune Checkpoint Inhibitors in Head and Neck Squamous Cell Carcinomas

**DOI:** 10.3389/fonc.2021.596290

**Published:** 2021-03-05

**Authors:** Lucas V. dos Santos, Carina M. Abrahão, William N. William

**Affiliations:** Centro de Oncologia, Hospital BP, A Beneficência Portuguesa de São Paulo, São Paulo, Brazil

**Keywords:** head and neck (H&N) cancer, immunotherapy, programmed death-1 (PD-1), programmed death ligand-1 (PD-L1), programmed death-1 (PD-1)/programmed death ligand-1 (PD-L1) axis, resistance

## Abstract

Preclinical data suggest that head and neck squamous cell carcinomas (HNSCC) may evade immune surveillance and induce immunosuppression. One mechanism of immune evasion involves the expression of programmed death ligand-1 (PD-L1) in tumor and immune cells, which is, to date, the only biomarker routinely used in clinical practice to select patients with advanced HNSCCs more likely to benefit from anti-PD-1 therapy. Nonetheless, PD-L1 expression alone incompletely captures the degree of sensitivity of HNSCCs to PD-1 inhibitors. Most patients exposed to anti-PD-1 antibodies do not respond to therapy, suggesting the existence of mechanisms of *de novo* resistance to immunotherapy. Furthermore, patients that initially respond to PD-1 inhibitors will eventually develop acquired resistance to immunotherapy through mechanisms that have not yet been completely elucidated. In this article, we will provide an overview of the immune landscape of HNSCCs. We will briefly describe the clinical activity of inhibitors of the PD-1/PD-L1 axis in this disease, as well as biomarkers of benefit from these agents that have been identified so far. We will review pre-clinical and clinical work in cancers in general, and in HNSCCs specifically, that have characterized the mechanisms of *de novo* and acquired resistance to immunotherapy. Lastly, we will provide insights into novel strategies under investigation to overcome resistance to immune checkpoint inhibitors.

## Introduction

In recent years, our understanding of the importance of the immune system and its interaction with tumor cells and tumor microenvironment has allowed us to explore an increasing number of immune modulation strategies for cancer therapies (1). The identification of so-called checkpoints in T-cell immunity—namely, the molecules programmed death-1 (PD-1) and cytotoxic T-lymphocyte-associated protein 4 (CTLA-4), as well as the development of function-blocking antibodies against these molecules, have paved the way towards our understanding of the relevance of the immune system against cancer and its manipulation.

Immunologic checkpoints are a complex homeostatic system of signaling pathways that mediate the activation or selective tolerance of the immune system towards target cells (2). These pathways serve to establish an effector response to non-self-antigens while preventing the induction of autoimmune activity. Tumor cells, including head and neck squamous cell carcinomas (HNSCCs), hijack these mechanisms of immunologic surveillance and control to create an immunosuppressive and protumor microenvironment. As a result, immunotherapy with PD-1 blockade has emerged as the latest standard-of-care treatment strategy developed for advanced HNSCCs.

In this article, we will provide an overview of the immune landscape of HNSCCs. We will briefly describe the clinical activity of inhibitors of the PD-1/PD-L1 axis in this disease, as well as biomarkers of benefit from these agents that have been identified so far. We will review pre-clinical and clinical work in cancers in general, and in HNSCCs specifically, that have characterized the mechanisms of *de novo* and acquired resistance to immunotherapy. Lastly, we will provide insights into novel strategies under investigation to overcome resistance to immune checkpoint inhibitors.

## Head and Neck Squamous Cell Carcinomas and the Cancer Immunity Cycle

HNSCC can evade immune surveillance through several crosslinked mechanisms that have now been recognized as being central to the development and progression of upper aerodigestive tract malignancies (3). The most clinically relevant mechanism of immune evasion identified so far is the modulation of cytotoxic T lymphocyte (CTL) activity.

A dual signal is mandatory for activation of CTLs against tumor antigens: the recognition of major histocompatibility complex (MHC)-antigen by the T cell receptor, and the interaction of B7 in the antigen presenting cell with CD28 in the CTL (4). This process primarily occurs in the lymph nodes and is regulated by immune checkpoint molecules. CTLA-4 is mostly expressed in CTLs, as well as in regulatory T lymphocytes (T regs). Upon binding to CTLA-4, the B7 protein induces CTL inhibition, and may cause CTL exhaustion (5). CTLA-4 expression is also upregulated by the immunosuppressive molecule transforming growth factor-β (TGF-β) produced by tumors cells (6). T regs are also one of the most important sources of TGF-β, thus contributing to T cell exhaustion (7, 8).

At the tissue level, T cell cytotoxicity is modulated by PD-1 and its ligands. PD-1 is a transmembrane protein belonging to the CD28 receptor family, which is highly expressed on T and B lymphocytes. The most important ligands for PD-1 include PD-ligand 1 and 2 (PD-L1 and PD-L2). These ligands are mostly expressed on antigen presenting cells, endothelial cells, as well as in CTLs (9, 10). Tregs inhibit CTLs by PD-1-PD-L1 interaction, thus promoting immunosuppression (11, 12). Hyperexpression of PD-1 in CTLs may also contribute to the immunosuppressive status due to enhanced sensitivity to PD-L1 (13). Like many other cancers, HNSCCs express PD-L1 on tumor cells, generating an immunosuppressive state and contributing to tumor progression and metastasis, with a negative impact on prognosis (14–16). Depending on the assay, PD-L1 is detected in about 50–70% of HNSCCs, and expression in Human Papilloma Virus (HPV)-related HNSCC is higher than in unrelated tumors (16). Indeed, HPV-related HNSCC are especially dependent on PD-L1 expression. HPV (+) tumors are characterized by more lymphocyte infiltration, with higher expression of PD-1 on CTLs when compared to HPV (−) tumors (17). In fact, at least three types of immune response in HPV (+) HNSCC have been described, rendering our understanding of the tumor host immune interactions more complex than previously thought (18).

In addition to CTLA-4 and PD-1 axes-mediated mechanisms, tumor immune evasion involves other related processes (19), selectively and briefly described as follows, some of which may represent therapeutic targets: (i) Neoangiogenesis induced by tumor associated macrophages exacerbates hypoxia and lowers the microenvironment pH, leading to PD-L1 upregulation and impairment of CTLs proliferation and efficiency (20–22); (ii) Chemokines and molecules, such as vascular endothelial growth factor (VEGF), interleukin 10 (IL-10), prostaglandin E2 and TGF-β, produced by Tregs and myeloid-derived suppressor cells (MDSCs), as well endothelial cells, reduce the attraction of CTLs (23). On the other hand, release of CXCL8, CCL2, CXCL5, and CXCL12, CCL22, and CCL28 attracts Tregs (24); (iii) Arrest of clonal expansion of CTLs, mediated by tumor cell, dendritic cell, and MDSCs secretion of indolamine-2,3-oxygenase (IDO), which induces degradation of tryptophan, an indispensable molecule for CTLs growth and production of Granzyme B (25); (iv) Impaired expression of human lymphocyte antigen-I (HLA-I) and other molecules involved in the antigen presentation machinery, leading to reduced tumor antigen recognition, impaired immune response, and worse prognosis (26, 27). Genetic alterations identified by The Cancer Genomic Atlas Network (TCGA), such as mutations in KMTD2 and HLA-A, contribute to this immunosuppressive behavior (28). Even though the complete loss of HLA-I could lead to T cell recognition evasion, activation of natural killer (NK) cells could take place, illustrating the potential for targeting multiple immune pathways for cancer therapy (29, 30).

## The Immune Landscape of Head and Neck Squamous Cell Carcinomas

Analyses of transcriptomics, genetic mutations, and copy number alterations in HNSCCs have revealed subtypes with common characteristics that may determine sensitivities to immunotherapies. Specifically, HPV (−) HNSCC may be subdivided into copy number high and low (28). Copy number low HPV (−) HNSCC, as well as HPV (+) HNSCC have been shown to have increased expression of immune signatures predictive of benefit from immune checkpoint inhibitors (31). Likewise, in a pan-TCGA analysis including HNSCC, lymphocyte infiltration correlated negatively with copy number variation segment burden, and positively with aneuploidy, loss of heterozygosity, homologous recombination deficiency, and tumor mutational burden (TMB) (32). In another pan-TCGA analysis, somatic copy number variation scores were positively correlated with mutations in driver genes involved in the DNA damage response pathway, as well as reduced cytotoxic immune infiltration—arm/chromosome somatic copy number variation scores were stronger predictors for decreased expression of immune signatures compared to focal copy number variation scores, including in HNSCCs with high TMB (33).

Recently, six immune subtypes across multiple tumor types were identified in an extensive pan-cancer TCGA immunogenomic analysis: wound healing, interferon-γ dominant, inflammatory, lymphocyte depleted, immunologically quiet, and TGF-β dominant. These tumors were characterized by differences in macrophage or lymphocyte signatures, Th1:Th2 cell ratio, intra-tumoral heterogeneity, copy number alterations, neoantigen load, cell proliferation, expression of immunomodulatory genes, and prognosis (34). The vast majority of squamous cell carcinomas were of the wound healing or interferon-γ dominant subtypes, with no significant differences in survival between these two groups. However, a more recent TCGA evaluation further stratified squamous cell carcinomas into six immune subtypes with distinct molecular characteristics and outcomes (35). The immune-cold subtype had the lowest level of T cell infiltration, the highest rate of aneuploidy, translating into worst survival. A subtype with M2-polarized macrophages, TGF-β signaling and reactive stroma also had a poor outcome compared to the other subtypes. The subgroup with the best survival rates was characterized by high CTLs and NK infiltration and elevated interferon-γ signature (35). In another study focusing specifically on HNSCCs, Chen et al. proposed three subgroups which were consistent with (albeit less granular than) the aforementioned analysis: immune active (enriched by proinflammatory M1 macrophage signature, with increased cytolytic activity and tumor infiltrating lymphocytes, and high incidence of HPV infection); immune exhausted (enriched by activated stroma and anti-inflammatory M2 macrophage signatures, with activation of the WNT/TGF-β signaling pathway activation and poor survival), and a non-immune class (36). In another TCGA comprehensive HNSCC immune landscape study, Mandal et al. demonstrated that both HPV (+) and HPV (−) HNSCCs were one of the most immune infiltrated tumors. However, most of the immune infiltrate was comprised of Tregs, which suppress immunological activities. NK population was also remarkably abundant in both subtypes of HNSCC (37). HPV-related HNSCC demonstrated the highest immune infiltration and increased cytolytic activity, which was counterbalanced by an increased Treg/CTLs ratio, whereas smoking related HNSCC had the lowest level of immune infiltration and interferon-γ signature. Patients with adaptative immune response cell infiltrates and mutations had improved survival when compared to those with innate immune response infiltrate and copy number alterations, suggesting a possible role for new immunotherapeutic approaches targeting Tregs and NK cells in improving efficacy of anti-PD-1 (37). Lastly, Cillo et al. assessed the transcriptional profiles of single cells from peripheral and intra-tumoral immune populations from patients with HPV (−) and HPV (+) HNSCCs and showed that helper CD4+ T cells and B cells were relatively divergent and CD8+ T cells and CD4+ regulatory T cells were relatively similar. They also identified a gene expression signature associated with CD4+ T follicular helper cells and longer progression-free survival (38).

The immune phenotype of HNSCC has also been characterized in terms of spatial distribution of tumor infiltrating lymphocytes. Troiano et al. classified tongue carcinomas into immune-inflamed (when lymphocytes were found next to tumor cells), immune-excluded (when lymphocytes were found in the stroma, outside the tumor), or immune-desert (absence of lymphocytes). Immune desert was the less frequent subgroup, but exhibited worse overall survival (39).

Taken together, these findings suggest a complex immune landscape, associated with (and possibly determined by) genomic alterations, with important implications to HNSCC prognosis. Interestingly, in one report, the transcriptomic variability of immunologic signatures seemed to be stable in both a spatially, and short-term, timely manner, minimizing the importance of tumor heterogeneity in selecting immunotherapeutic approaches, at least for untreated patients (40). The data provide rationale for development of PD-1 inhibitors for HNSCCs along with potential biomarkers of efficacy, and for development of combination immunotherapeutic approaches for management of patients harboring tumors with *de novo* and/or acquired resistance to such immunotherapies.

## Standard Immunotherapies for Head and Neck Squamous Cell Carcinomas

The identification that lymphocytes could take part in the immune response in cancers (including HNSCCs) was identified several years ago (41), and evolved to the development of cancer immunotherapy, initially for melanomas, later extended to HNSCCs and other tumor types. Simplistically, the ultimate goal of immunotherapy is to relieve immunosuppression, and thus induce responses in tumor, without auto-immune adverse events (42, 43). To date, the anti-PD-1 antibodies nivolumab and pembrolizumab have been investigated in phase 3 studies and are the only immunotherapies approved by regulatory agencies worldwide for treatment of advanced HNSCCs.

In patients with recurrent or metastatic HNSCC that failed platinum-based chemotherapy enrolled in the Checkmate-141 study, nivolumab, when compared to the investigators’ choice single agent therapy, improved overall survival (OS) and overall response rate (ORR). Importantly, the rate of grade 3–4 adverse events was lower with nivolumab than chemotherapy (44). In the KEYNOTE-040 study with analogous design, pembrolizumab demonstrated similar benefits against investigators’ choice standard therapy, although statistical significance for the primary OS endpoint was not reached (45).

These encouraging results led to the development of pembrolizumab in the first-line setting for recurrent/metastatic disease. In the KEYNOTE-048 study, with a complex statistical design and assumptions, pembrolizumab either as monotherapy or in combination with cisplatin or carboplatin and 5-fluorouracil (5-FU) was compared to platinum plus 5-FU combined with cetuximab (EXTREME regimen) in patients that had failed curative-intent therapies including surgery and/or radiation therapy (46). Compared to the EXTREME regimen, pembrolizumab monotherapy improved OS in the PD-L1 combined positive score (CPS) ≥ 20 or CPS ≥ 1 populations. The safety profile was improved in the pembrolizumab arm. Data on the chemotherapy plus pembrolizumab cohort is discussed below [see PD-(L)1 inhibitors plus chemotherapy subsection]. These results led to the FDA-approval of pembrolizumab as first-line monotherapy in patients with recurrent or metastatic disease with PD-L1 CPS ≥1, and in combination with chemotherapy independently of PD-L1 expression (47). However, the European regulatory agency recommended the approval of first-line pembrolizumab (whether monotherapy or in combination with platinum and 5-FU) only in patients with PD-L1 expressing tumors (CPS of 1 or above) (48). Nonetheless, PD-L1 expression alone incompletely captured the degree of sensitivity of HNSCCs to PD-1 inhibitors. Most patients exposed to anti-PD-1 antibodies did not respond to therapy, suggesting the existence of mechanisms of *de novo* resistance to immunotherapy. Furthermore, most patients that initially respond to PD-1 inhibitors eventually develop acquired resistance to immunotherapy through mechanisms that have not yet been completely elucidated. These data illustrate the need to discover more accurate biomarkers of sensitivity to PD-1 axis blockade, as well as strategies to enhance activity of and/or overcome resistance to these drugs.

## Predictive Factors for Immunotherapy Benefits

Both PD-L1 and PD-L2 expression have been reported in many tumor types (including HNSCCs) and were amongst the first candidate biomarkers of immunotherapy efficacy investigated across several trials (49–51). Several PD-L1 assays are available in oncology, and they seem to be highly interchangeable in HNSCC (52), specially for assays evaluating tumor cells by the antibodies SP263, 22C3, and 28-8 (53). Concordance between staining scores that involve immune cells, and/or other antibodies (e.g., SP142) are more modest (53, 54) and require more careful interpretation. In the pivotal phase 3 Checkmate-141 trial, OS and ORR were improved by nivolumab across the entire study population. However, the magnitude of benefit seemed higher in patients with PD-L1 expression in at least 1% of the tumor cells using the 28-8 assay. HPV (+) and HPV (−) cancers derived the same benefit from nivolumab. No interaction between HPV and PD-L1 status was observed in this clinical trial (44, 55). Pembrolizumab was first explored in advanced HNSCC in the multi-cohort phase Ib KEYNOTE-012 trial (56). Anti-PD-L1 22C3 and anti-PD-L2 3G2 antibodies were used for PD-L1 and PD-L2 immunohistochemical assays, respectively. Overall, a 4% complete response (CR) and 14% partial response (PR) rate was observed, and 60% of patients experienced reductions in target lesions. PD-L1 CPS (which takes into account PD-L1 expression in both tumor and immune cells) performed better than the tumor proportion score (TPS) in predicting response to pembrolizumab, emerging as the most reliable biomarker for pembrolizumab. PD-L2 and PD-L1 expression were correlated, and PD-L2 expression was also associated with higher ORR. Patients with co-expression of PD-L1 and PD-L2 had higher ORR compared to PD-L1 positive patients alone. However, a 9% ORR was found in patients without the expression of any biomarker, underscoring the limitations of these strategies in selecting patients for pembrolizumab therapy (56). Similar results were found in the single arm, phase 2 KEYNOTE-055 study (57). These data supported the incorporation of PD-L1 expression (assessed by CPS) into the statistical design of the first-line KEYNOTE-048 study, as previously described.

TMB has been postulated as a possible biomarker of immunotherapy efficacy in cancers. Presumably, high TMB increases the abundance of neo-antigens (or neo-epitopes) resulting from non-synonymous mutations on cancer cells, allowing immune recognition and specific CTLs activation (58–60). However, only a small number of missense mutations produce neo-antigens, and a smaller part of those neo-antigens ultimately are recognizable by CTLs (61, 62). As such, specific immunogenic mutations, rather than total mutational burden, may be associated with improved prognosis, as it leads to increased expression of CD8A and hyperexpression of PD-1 and CTLA-4 (61). Despite these limitations, TMB has been associated with improved outcomes in clinical trials. In KEYNOTE-012, using a cut off ≥ 102 mutations per exome, TMB was associated with improved ORR (63–65). In a series of 126 patients treated at the Dana Farber Cancer Institute, TMB was higher in former smokers compared to non-smokers and HPV (+) patients, as well as in responders. Among HPV (−) responders, NOTCH1 and SMARCA4 were more frequently mutated, and frameshift events in tumor suppressor genes occurred more frequently. T cell immunoglobulin mucin-3 (TIM-3)/lymphocyte activated gene-3 (LAG-3) co-expression with PD-1 was higher on T cells among non-responders, suggesting a possible mechanism of adaptive *de novo* immune resistance (66). Consistent with the KEYNOTE-012 and Dana Farber data, a *post hoc* analysis of the EAGLE study revealed that a blood TMB of 16 mutations/Mb was associated with improved OS in the second-line setting for the anti-PD-L1 durvalumab alone, or in combination with the anti-CTLA4 tremelimumab versus single agent chemotherapy (67). Of note, blood TMB seemed also to be prognostic in the EAGLE study, since median OS in the standard of care arm was 4.0 months for high TMB versus 8.6 months for low TMB, raising the question whether this biomarker could be associated with poor outcomes to chemotherapy alone (67).

Interferon-γ and its co-stimulatory chemokines are implicated in tumor innate immune sensing, leading to immediate CTLs recruitment into the tumor micro ambient, a key step for an effective immune response (68–70). Interferon-γ gene expression was associated with clinical response in several cancer types treated with pembrolizumab (71). Likewise, in the KEYNOTE-012 study, a 6-gene interferon-γ signature including IDO1, CXCL10, CXCL9, HLA-DRA, STAT1, IFN-γ gene expression was found to be associated with improved ORR or progression-free survival (65).

A major role of fecal microbiome in determining response to immunotherapy has been increasingly recognized in recent years (72). Several mechanisms have been implicated in the dynamic interaction between microbiome and immunologic response, including T-cell activation, influence on recognition pattern of antigens (73). Some specific bacterial genera have been identified as predictors of response and toxicity in fecal microbiota transplant (FMT) experiments in mice. *Akkermansia muciniphila* was associated with increased response to anti-PD-1 (74). *A. muciniphila*, and *Enterococcus hirae* was able to reverse resistance to immunotherapy in mice. The mechanism implicated in such effects were related to increase in CCR9, CXCR3, and promotion of CTLs infiltration (75). Studies in melanoma suggested that the presence of certain genera, like *Bifidobacterium longum*, *Collinsella aerofaciens*, *Enterococcus faecius*, *Faecalibacterium spp* and *Ruminococcaceae spp* were associated with increased response to immunotherapy, and *Bacteriodales* were more common among non-responders. Responders were more likely to harbor greater microbiome diversity than non-responders (76, 77). Recently, data from phase 3 randomized trials comparing anti-PD-1 to chemotherapy showed that the use of antibiotics impaired the OS of patients receiving anti-PD-1 without compromising survival in the control group, suggesting a major role of microbiota in the benefit of immunotherapy in HNSCC (78). Oral cavity microbiome has also been implicated in HNSCC carcinogenesis and progression. Usually, *Fusobacteria* are abundant in primary and metastatic tissues, whereas *Streptococcus* have limited homing (79). Smoking and alcohol consumption are major risk factors for both HNSCC and periodontal disease, and are key modifiers of oral microbiota (80, 81). Abundant *F. periodonticum* and *S. mitis* and *P. pasteri* paucity are associated with late stage oral cancer (82). Despite these promising data, oral microbiome was not associated with outcomes in the Checkmate 141 study (83). Evaluation of fecal specimens may better reflect patients’ microbiome, but have not been assessed in anti-PD-1 trials in HNSCCs.

Taken together, these data demonstrate that a robust biomarker of sensitivity to PD-1/PD-L1 blockade has yet to be developed. It is likely that multiple mechanisms of resistance to immunotherapies are in place, leading to low response rates to single agent PD-1 inhibitors in all HNSCC clinical trials performed to date. Few comprehensive upfront and re-biopsy studies for biomarker evaluation (especially upon disease progression) have been completed. As will be discussed below, such investigations would be essential for the rational design of strategies aiming at mitigating resistance to treatment.

## Resistance to Immunotherapies

From a clinical perspective, resistance to immunotherapy may be divided into *de novo* or acquired. *De novo* (or primary) resistance may be defined as lack of benefit from upfront immunotherapy treatment, whereas acquired (or secondary) resistance is characterized by an initial period of benefit from immunotherapy followed by disease progression. Mechanistically, *de novo* and acquired resistance to immunotherapy may share common processes, including adaptive immune resistance (whereby the cancer is recognized by, but evades the immune system, by adapting to the immune attack). Additionally, acquired resistance to immunotherapy may emerge from adaptive resistance that occurs in a relatively homogenous fashion, and/or by selection of heterogenous clones over time that were already resistant to immunotherapy, even before treatment initiation (84).

Mechanisms of resistance to immunotherapy include tumor cell-intrinsic and tumor cell- extrinsic factors. Tumor cell-intrinsic resistance may stem from absence of antigenic proteins, absence of antigen presentation, genetic T cell exclusion, and/or insensitivity to CTLs. Tumor cell-extrinsic resistance may be a result of absence of CTLs, expression of inhibitory immune checkpoints, and/or presence of immunosuppressive cells (84–86). None of these mechanisms have been extensively studied in HNSCCs, limiting our understanding about the dynamic pressures on the immune system at play upon immunotherapy administration, and hindering our ability to rationally design combination and/or sequential approaches to mitigate resistance to PD-1 inhibitors. Knowledge gained from other cancers and pre-clinical work might prove to be relevant to HNSCC patients and is therefore described below.

Beta-2-microglobulin (B2M) has an important function on HLA class I transport to cell membrane, and inactivating mutations in B2M lead to loss of expression of HLA class I, impairing immune response (87, 88). This mechanism of resistance to anti-PD1 in metastatic melanoma has been detailed elsewhere (89). Other groups reported that B2M mutation in other clinical settings could also lead to acquired resistance to immunotherapy (90–92). Other causes of HLA class I loss of expression with intact B2M may also induce disease progression to anti-PD1 (90). Saloura et al. have demonstrated more diverse T-cell repertoire in HPV (+) versus (−) HNSCC, possibly due to impaired HLA class I expression induced by the virus (93). As such, strategies that could restore HLA class I expression could potentially be developed to augment immune response in this setting.

Release of IFN-γ by CTLs may induce PD-L1 and MHC class I expression in tumor cells through activation of the JAK-STAT pathway. Several mechanisms of tumor cell death derive from this pathway (94). Clinical evidence recognizes that mutations in JAK1 and JAK2 can be responsible for the progression of metastatic melanoma after initial response to anti-PD1 (89). It is unknown whether alterations in other molecules in the JAK-STAT pathway could be implicated in acquired resistance to immunotherapy, but their role in primary resistance has already been demonstrated (95).

The loss of mutations that preclude the expression of neoantigens recognized by the immune system through clonal selection, copy-number loss, or epigenetic mutation may lead to immune evasion and clinical progression (96). In a case series of four patients with non-small cell lung cancers, mutations encoding neoantigens were lost, and progressive disease occurred after initial response do anti-PD1 therapy (97) Clonal pressure has been implicated in immunoselection of tumor cells that respond to CTLs and adoptive cell transfer immunotherapy (98–100).

Phosphatase and tensin homolog on chromosome 10 (PTEN) inactivation and, consequently, phosphoinositide 3-kinase (PI3K) pathway activation is related to an immunosuppressive tumor microenvironment that may have implications in resistance to immunotherapy (101). A total of 55 isocitrate dehydrogenase 1 wild-type glioblastoma patients who received immunotherapy, including 13 long-term responders, were analyzed in one report, and PTEN mutations were identified in 23 out of 32 non-responders, but only in 3 responders (102). PTEN mutations were also associated with an immunosuppressive signature. Similar results were found in non-small cell lung cancer and melanoma patients, indicating a putative effect of PTEN-loss in acquired resistance to immunotherapy (103, 104). PTEN-loss may also be implicated in secondary resistance to immunotherapy in other distinct tumor types (105, 106). Patients with metastatic melanoma who initially responded to anti-PD-1 alone or in combination with anti-CTLA-4 and then progressed were analyzed, and PTEN-loss was identified in 5 cases in the post-progression biopsy out of 18 intact PTEN expression in pre-treatment biopsies (107). In surgically treated oral cavity squamous cell carcinomas, PTEN loss in tumor infiltrating immune cells has been associated with worse prognosis (108). Similarly to PTEN-loss, WNT-β-catenin promotes an immunosuppressive tumor microenvironment that may be responsible for secondary resistance to immunotherapy (106, 109).

Modulation of other immune checkpoints has been identified in patients with secondary resistance to immunotherapy, including, but not limited to, TIM-3, LAG3, and V-domain immunoglobulin suppressor of T cell activation (VISTA), glucocorticoid induced TNFR family related gene (GITR), and T cell immunoglobulin and immunossupressor tyrosine kinase-based inhibitory motif (TIGIT) (90, 107, 110).

TIM-3 is a member of TIM family expressed on CD4(+) Th1 but not Th2 lymphocytes (111). It is also expressed in tumor cells and other immune cells (112). TIM-3 and its ligands, such as galectin-9, may regulate several biological functions of tumor cells, including aggregation, adhesion and apoptosis (113, 114). The binding of TIM-3 to galectin-9 leads to promotion of apoptosis of Th1 cells, impairs function of CTLs and induces major expansion of MDSCs, suppressing immune response. In early stages of disease, TIM-3 may have an immunostimulatory effect favoring CTLs secretion of interferon-γ, but TIM-3 expression in Tregs in late-stage tumors favor the suppression of CTLs and are important to create an immunosuppressive environment. Anti-TIM-3 monoclonal antibody may suppress the inhibition of CTLs and improve antitumor response (115). TIM-3 expression has been implicated in nodal metastasis and recurrence in HNSCC (116). TIM-3 may be related to the exhaustion of CTLs and ineffective immune response in HNSCC, favoring metastatic behavior (117) In a HNSCC mouse model, anti-TIM-3 antibody induced activation of CTLs and suppressed MDSCs, inhibiting carcinogenesis and improving antitumor responses (116).

LAG3, also known as CD223, is mainly expressed in activated T cells and, to a lesser extent, NK cells, B cells and dendritic cells. LAG-3 reduces T cell proliferation and activation (118, 119). LAG-3 is also an effector of Tregs inhibitory function (120). Tumor-infiltrating lymphocytes co-expressing PD-1 and LAG-3 may be susceptible to inhibition, leading to immune scape of cancer cells (121). LAG-3 also binds to liver and lymph node sinusoidal endothelial cell C-type lectin (LSECtin) and inhibit the secretion of interferon-γ by CTLs, therefore inhibiting immune response (122). Fibrinogen-like protein 1 (FGL1) is a liver secreted protein which inhibits the activation of T cells (123, 124). FGL1 is upregulated in several human cancers and it is associated with impaired outcome and blocking of FGL1-LAG-3 interaction enhances T cell response and improves antitumor immunity (123, 124). In HNSCCs, LAG-3 overexpression is associated with worse prognosis, and LAG-3 blockade retarded tumor growth in a HNSCC mouse model (125).

VISTA is another checkpoint similar in function to PD-L1 and capable of suppressing T effector cells. VISTA is expressed on myeloid APCs and Tregs (126). VISTA enhances Treg maturation and suppresses T cell activation (127). V-set and immunoglobulin domain-containing 3 (VSIG3) interacts with VISTA on activated T cells, suppress T cell proliferation and induces the production of immunosuppressive cytokines and chemokines. Data from several tumor types support blocking of VSIG3/VISTA pathway as a promising immunotherapy strategy (128). In HNSCC patients, overall survival was reduced when VISTA expression was high simultaneously with low CD8+ infiltration (129).

GITR is expressed on the surface of CD25+CD4+ Tregs, CTLS and NK cells (130). Binding of GITR to its ligand GITRL may impair the attraction of Tregs, weaken their suppression activity and activate the MAPK (mitogen-activated protein kinase)/ERK pathway and NF-κB signaling, which ultimately induces T cell proliferation and pro-inflammatory cytokines (131–133).

TIGIT is expressed mainly in effector lymphocytes and NK cells (134). CD155 is highly expressed in tumor cells and has high affinity to TIGIT, and induces IL-10 secretion, reduces the secretion of pro-inflammatory cytokines and inhibits antitumor response (135). TIGIT shares the same ligands with CD226, which, in part, counterbalances the TIGIT immunosuppressive effect (136). In mouse models of HNSCCs, TIGIT blockade delayed tumor progression through mechanisms involving CD8+ CTLs activation and Tregs inhibition. PD-1/PD-L1 inhibition increased expression of TIGIT on Tregs (137).

Some mechanisms of resistance may be induced by previous treatment. For example, in the Checkmate-064 study, patients with metastatic melanomas were randomized to ipilimumab followed by nivolumab after 12 weeks or the opposite order, and the immune landscape was analyzed at baseline and at week 13. Some immunophenotypes were more prone to show responses to ipilimumab and progression to nivolumab, and *vice versa*. Ipilimumab and nivolumab induced different patterns or immune landscape change after 12 weeks, and such patterns were related to patient outcomes. Furthermore, the alterations induced by ipilimumab favored progression in the nivolumab-ipilimumab cohort, whereas the alterations induced by nivolumab favored response in the nivolumab-ipilimumab cohort (138). These findings explain the superior survival outcomes in the nivolumab-ipilimumab arm (139).

## Discussion and Future Directions

**Figure 1:**
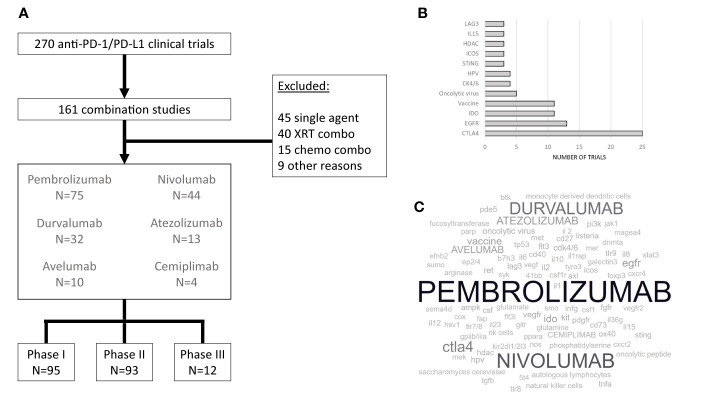
Clinical trials evaluating immunotherapy in HNSCC. **(A)** Completed, ongoing, or terminated HNSCC clinical trials involving pembrolizumab, nivolumab, durvalumab, atezolizumab, avelumab, or cemiplimab as single agents or in combination with drugs other than cytotoxic chemotherapy. **(B)**. Most common co-targets (or mechanism of action, when appropriate – e.g. oncolytic virus) for anti-PD-1 or anti-PD-L1 combinations **(C)**. A word cloud visual representation of PD-1 or PD-L1 inhibitors (in capital letters) and their co-targets (in small caps) under evaluation in combination studies. The font size is proportional to the number of studies employing the intervention.

There is an intricate interplay between the immune system, other components of the tumor microenvironment, and cancer cells that ultimately contribute to carcinogenesis and determine sensitivity and resistance to therapeutic strategies that have been developed so far to manage HNSCCs. The complexities of the microenvironment-cancer cell equilibrium outlined above in this review suggest that single-agent anti-PD-1/PD-L1 therapy would not be sufficient to promote long-term disease control. A natural evolution in the clinical development process of pharmacologic agents to treat HNSCCs would be the study of drug combinations, many of which have not been thoroughly investigated in pre-clinical systems specific to head and neck cancers but are already undergoing testing in human trials. This rapid pace of clinical investigations underscores a new model of information “cross pollination” from one cancer type to the next that, on the one hand, could reduce the likelihood of success of each individual study (given less robust rationale), but on the other hand may collectively result in identification of improved treatment options for patients with malignant diseases that were previously considered low priority for drug development, such as HNSCCs. Indeed, at the time of this writing, a search on *clinicaltrials.gov* using the terms “head and neck cancer” AND “nivolumab”, “pembrolizumab”, “durvalumab”, “atezolizumab”, “avelumab”, OR “cemiplimab” (i.e., PD-1/PD-L1 inhibitors already approved for at least one cancer type) resulted in 270 studies (**Figure 1**). Below, we briefly discuss all phase 3 drug combination trials identified, whether ongoing, completed or terminated.

### Programmed Death Ligand-1 Inhibitors Plus Chemotherapy

Chemotherapy has been proposed as a combination strategy to enhance immunotherapy efficacy and bypass *de novo* and/or acquired resistance to PD-(L)1 inhibitors, through mechanisms that might involve increase of mutational load in cancer cells, depletion of suppressive regulatory T cells and myeloid-derived cells, normalization of neovasculature (thus facilitating T cell infiltration), upregulation of HLA class I expression and other components of antigen presentation machinery, induction of immunogenic cancer cell death (leading to neoantigen cross presentation), and modulation of cell signaling to increase sensitivity to interferon-gamma (140). In the KEYNOTE-048 phase 3 study, platinum, 5-FU plus pembrolizumab was compared to platinum, 5-FU plus cetuximab, and results demonstrated an improvement in overall survival for patients with PD-L1 expression CPS ≥ 20, CPS ≥ 1, or in the total population (regardless of PD-L1 expression) (46). Post-hoc analysis has demonstrated that efficacy improvement for the combination was primarily restricted to the PD-L1 positive group (141), raising the possibility that chemotherapy may not contribute to overcoming resistance to immunotherapy in patients without PD-L1 expression. A comparison between the pembrolizumab and chemotherapy plus pembrolizumab arms was not planned per trial design, and therefore it remains to be determined whether the long-term benefits from treatment in the pembrolizumab-containing arms can be attributed to the immunotherapy alone, or may be a result of a synergistic effect between chemotherapy and immunotherapy contributing to mitigation of resistance to treatment. Several chemotherapy plus immunotherapy trials are under way (**Figure 1**), combined or not with radiation therapy (see below).

### Programmed Death Ligand-1 Inhibitors Plus Radiation Therapy

The tumor immune microenvironment is dynamic and has been shown to be depleted of CD8+ T cells and B lymphocytes in recurrent versus primary tumors, with immune suppressive features apparent after receipt of chemoradiation therapy (142). Clonal expansion of tumor-infiltrating T cells has been identified in patients with untreated, locoregionally advanced SCCHN (93). These observations increase the enthusiasm for incorporating immunotherapy for earlier stages of HNSCC, in which immune function seems to be better preserved, and for which radiation therapy-based strategies are often used as a standard of care. Adiotherapy-induced immunosuppression has been well characterized (143). In animal models, PD-L1 blockade combined with radiation therapy reverses T cell exhaustion and leads to oligoclonal T cell expansion (144), suggesting a possible role of PD-(L)1 inhibitors in contributing to disease control in this setting. Radiation therapy may also synergize with immune checkpoint inhibitors through other nonredundant pathways that enhance antitumor activity, reviewed elsewhere (143, 145). Results of the first randomized studies combining PD-(L)1 inhibitors with radiation therapy for locally advanced HNSCC have recently been presented. In the GORTEC 2015-01 PembroRad trial, pembrolizumab plus radiation therapy failed to improve locoregional control compared to cetuximab plus radiation therapy in patients unfit for platinum (146). Likewise, in the phase 3 JAVELIN Head and Neck 100 trial, addition of avelumab to cisplatin/radiation therapy did not improve progression-free or overall survival (147). In advanced disease, addition of stereotactic radiation therapy to nivolumab has also been evaluated as a strategy to induce abscopal effect in a randomized phase 2 trial. Unfortunately, there were no improvements in overall response rates of nonirradiated lesions (primary endpoint), progression-free or overall survival (148). Despite these early negative results, several studies continue to evaluate immunotherapy in the context of radiation therapy (**Figure 1**) and will eventually determine whether PD-(L)1 inhibitors and radiation therapy can be combined to effectively circumvent resistance to treatment.

## Programmed Death Ligand-1 Plus Cytotoxic T-Lymphocyte-Associated Protein 4Inhibitors

CTLA-4 was the first modern immunotherapy strategy to be widely explored in oncology. CTLA-4 can bind to B7, precluding the interaction between B7 and the co-stimulatory molecule CD28 and limiting the proliferation of T cells and the release of interleukin-2 (149). Blocking of CTLA-4 may limit the inhibitory effect on CTLs favoring host immune response. Due to its limited efficacy in other tumor types than melanoma, including HNSCC, anti-CTLA-4 has been developed mostly in combination with other agents (150–155). More recently, building on the results of the phase 2 CONDOR study (154), durvalumab, alone or in combination with tremelimumab, were compared to investigator’s choice chemotherapy (cetuximab, taxane, methotrexate, or fluoropyrimidine) in the phase 3 EAGLE study involving patients with HNSCC whose disease failed platinum-based chemotherapy. Durvalumab alone or in combination did not meet the primary outcome of OS benefit. Duration of response and 2-year survival were improved in the durvalumab monotherapy arm, suggesting that this drug is active in HNSCCs (156). PD-L1 expression, as assessed by SP263 assay, did not impact on any of the efficacy results, but a small benefit was found in patients with high TMB (≥16mutations/Mb) (67, 156). Despite these disappointing results, durvalumab alone or in combination with tremelimumab was evaluated in the first line setting in comparison with the EXTREME regimen in the KESTREL trial (NCT02551159), and results are pending. Nivolumab has also been tested in combination with ipilimumab versus nivolumab alone in the Checkmate-714 trial, and although the data have not yet been formally reported, a press release dated April 25, 2019 has indicated that the study did not meet its primary endpoint. In Checkmate-651 nivolumab plus ipilimumab has been compared against the EXTREME regimen (NCT02741570). Recruitment has already been completed, and the main data for these trials are expected in the following months. Nivolumab plus ipilimumab is also under development in the setting of locally advanced, potentially curable disease (NCT03700905). Taken together, the strategy of targeting PD-(L)1 plus CTLA-4 has not yielded promising results so far in phase 2/3 trials (154, 156). Unless the recently completed studies (NCT02551159, NCT02741570) report superior outcomes in the near future, other immunotherapy-immunotherapy combinations may need to be explored, as discussed below.

## Programmed Death-1 Plus Indolamine-2,3-Oxygenase Inhibitors

IDO is an enzyme that metabolizes tryptophan, limiting CTLs cytotoxicity. It is highly expressed in tumor-cells, macrophages and dendritic cells (157). Tryptophan depletion and its inhibitory metabolites has been implicated on how IDO is responsible for T cell anergy and suppression, as well as Treg activation and MDSCs infiltration (158, 159). IDO activity has been implicated on resistance to anti-PD-1 therapy (160, 161). Epacadostat, an IDO inhibitor, was evaluated in combination with pembrolizumab in advanced solid tumors, including HNSCCs. In phase I study ECHO-202/KEYNOTE-037, the ORR for this combination was 55%, including 13% complete responders. Two patients with refractory HNSCC were included, and one achieved a partial response and the other had stable disease with minor reduction in tumor burden (162). This study had a phase II part, including 36 additional HNSCC patients. The ORR was 30.5%, which was lower in patients with 3 or more lines of treatment (163). Nivolumab was also combined with epacadostat, with an ORR of 22.6% (164). Despite these results, its development as adjunctive therapy to anti-PD-1 in HNSCC (NCT03358472, NCT03342352) was halted due to the negative results of the combination IDO inhibitor and pembrolizumab in melanoma (165).

## Programmed Death-1 Plus B7H3 Inhibitors

B7 constitutes a superfamily of inhibitory molecules in the cancer microenvironment was highly related to the immune evasion of cancer cells (4). B7-H3 (also known as CD276) is a newly identified member of the B7 family (166, 167), which is found in several human cancer cells and APCs. B7-H3 induces proliferation of both CD4+ and CD8+ T cells, enhances CTLs, and stimulates IFN-γ production in the presence of T cell receptor signaling (167). B7-H3 was later found to negatively regulate T cell function, affecting preferentially T helper type 1-mediated immune responses (168). Overexpression of B7-H3 was associated with larger tumor, advanced stage, and impaired survival in oral cancer patients (169). Retifanlimab, an anti-PD-1 antibody, and enoblituzumab, an anti-CD276, were evaluated in combination in multiple tumor types cohorts. In the anti-PD-1 naïve HNSCC cohort, 18 patients were treated, and ORR were 33.3%, with five partial responders and one complete responder (170). This led to the subsequent development of a phase II/III study (NCT04129320).

## Programmed Death Ligand-1 Plus EGFR Inhibitors

As described in **Table 1**, a number of phase 2 studies have been completed targeting the PD-1/PD-L1 axis and EGFR in recurrent/metastatic disease. A randomized, phase 3 study is currently ongoing evaluating the role of avelumab added to concurrent cetuximab/radiation therapy in locally advanced HNSCCs. GORTEC-2017-01 is a two-cohort prospective clinical trial enrolling treatment naïve patients with resectable stage III-IVa HNSCC. Cisplatin-eligible patients will be randomized to radiation therapy plus cisplatin or radiation therapy plus cetuximab plus avelumab. Those who are unfit for cisplatin therapy will be randomized to radiation therapy plus cetuximab or radiation therapy plus cetuximab plus avelumab (NCT02999087).

**Table 1:** Select Phase I and II drug combination clinical trials with PD-1/PD-L1 inhibitors.

Drugs	PD-1/PD-L1 co-target	Summary of results	Reference
avelumab/cetuximab	EGFR	Phase-I feasibility trial evaluating RT plus cetuximab-avelumab in cisplatin-ineligible advanced HNSCC. 8/10 completed therapy. No grade 4-5 toxicity was found. PFS was 10mo. (NCT02938273)	Elbers et al. (171)
durvalumab/cetuximab	EGFR	Phase II clinical trial evaluating cetuximab plus durvalumab in advanced/metastatic HNSCC. The first preliminary report identified an activation of NK cell immune response. (NCT03691714)	Gulati et al. (172)
Pembrolizumab/cetuximab	EGFR	Multi-cohort phase II trial evaluating cetuximab plus pembrolizumab in advanced/metastatic, cisplatin-refractory or cisplatin-ineligible HNSCC patients. In anti-PD-1/cetuximab naïve patients cohort, 33 subjects were enrolled. ORR was 41% and PFS was 8.2mo. (NCT03082534)	Sacco et al. (173)
nivolumab/cetuximab	EGFR	Phase II clinical trial evaluating cetuximab plus nivolumab in previously treated advanced/metastatic HNSCC patients. Median PFS and OS were 3.4 and 11.5 months, respectively. (NCT03370276)	Chung et al. (174)
durvalumab/MEDI0457	HPV	Phase II window of opportunity trial evaluating durvalumab plus MEDI0457, a therapeutic vaccine against HPV, in potentially curable p16+ HNSCC patients. Only 3 out of 21 patients recurred. (NCT02163057)	Aggarwal et al. (175)
avelumab/TG4001	HPV	Phase Ib window of opportunity trial evaluating avelumab plus TG4001, a therapeutic vaccine against HPV, in advanced/metastatic HPV+ patients. T cell responses were observed. Three out of nine patients (including 5 with HNSCC) showed partial responses. (NCT03260023)	Le Tourneau et al. (176)
nivolumab/ISA101	HPV	Phase II clinical trial evaluating nivolumab plus ISA101, a therapeutic vaccine against HPV-16, in patients with advanced/metastatic HPV-16 positive cancers. Twenty two out of 24 patients had HPV+ oropharyngeal cancer. The ORR was 33.3%, and median PFS and OS were 2.7 and 17.5 months, respectively. (NCT02426892)	Massarelli et al. (177)
pembrolizumab/enoblituzumab	B7H3	Phase I clinical trial evaluating pembrolizumab and enoblituzumab in solid tumors. The ORR was 33.3% in 18 patients with HNSCC not previously exposed to anti-PD-1. (NCT02475213)	Aggarwal et al. (170)
pembrolizumab/eftilagimod alpha	LAG3	Phase II multi-cohort trial evaluating pembrolizumab plus eftilagimod alpha in lung and HNSCC patients. The ORR in HNSCC were 40% in 15 patients. (NCT03625323)	Felip et al. (178)
nivolumab/lirilumab	KIR2DL1/2L3	Phase I/II trial evaluating nivolumab plus lirilumab in advanced/metastatic HNSCC patients. Amongst 41 patients, the ORR was 24.1%, including 10.3% complete responders.	Leidner et al. (179)
atezolizumab/varlilumab	CD27	Phase I trial evaluating atezolizumab plus varlilumab in solid tumors. A total of 36 patients with solid tumors were included. Amongst three HNSCC patients, one responded. (NCT02335918)	Sanborn et al. (180)
pembrolizumab/SD-101	TLR9	Phase Ib/II trial evaluating pembrolizumab plus SD-101 in patients with advanced/metastatic HNSCC. Twenty-three patients out of 28 were evaluable for efficacy. The ORR was 22%, including two patients with complete response. (NCT02521870).	Cohen et al. (181)
durvalumab/AZD9150 durvalumab/AZD5069	STAT3 CXCR2	Phase Ib/II trial evaluating durvalumab in combination with AZD9150 or AZD5069 in anti-PD-1/PD-L1 naïve patients with advanced/metastatic HNSCC. In 38 patients in the AZD9150 cohort, ORR was 26%, including 4 complete responders. In 20 patients of AZD5069 cohort, ORR was 10%. (NCT02499328)	Cohen et al. (182)
pembrolizumab/GR-MD-02	Galectin3	Phase Ib trial evaluating pembrolizumab plus GR-MD-02 in patients with malignant melanoma, lung cancer and HNSCC. The ORR was 33% in the six HNSCC treated patients. (NCT02575404)	Curti (183)
pembrolizumab/INCB001158	Arginase	Multi-cohort phase I/II trial evaluating INCB00158 alone or in combination with pembrolizumab in advanced/metastatic solid tumors. Mature data for MSS CRC showed 28% ORR. HNSCC is yet to be reported. (NCT02903914)	Naing et al. (184)
durvalumab/metformin	AMPK	Phase I window of opportunity trial evaluating durvalumab plus metformin in patients with operable HNSCC. The combination was safe. Data on response is pending. (NCT03618654)	Richa et al. (185)
nivolumab/tadalafil	PDE5	Phase II window of opportunity trial evaluating nivolumab alone or in combination with tadalafil in patients with operable HNSCC. Patients (N=47) were randomized to nivolumab alone or in combination. Half of the patients responded, including 9% complete response rate. Tadalafil improved T cell infiltration. (NCT03238365)	Luginbuhl et al. (186)
durvalumab/olaparib	PARP	Phase II window of opportunity trial randomized operable HNSCC patients to cisplatin/olaparib, olaparib alone, no treatment or olaparib plus durvalumab. Two patients out of 11 responded to Olaparib-durvalumab, including a complete responder.	Psyrri et al. (187)

RT, Radiation Therapy; HNSCC, Head and neck squamous cell carcinoma; PFS, Progression-free survival; ORR, Objective Response Rate; HPV, Human Papilloma Virus; MSS, Microsatellite stable; CRC, Colorectal Cancer.

## Programmed Death-1 Inhibitor Plus Lenvatinib

As mentioned above, angiogenesis is closely related to immune response and may take part in the development of *de novo* or acquired resistance to immunotherapy. Lenvatinib is a multi-kinase inhibitor of vascular endothelial growth factor receptors 1–3, was combined with pembrolizumab in a phase Ib/II clinical trial. The ORR was 36.4% in 22 evaluated HNSCC patients (188). These results lead to the development of a placebo-controlled randomized phase 3 trial enrolling patients with HNSCC with no prior therapy for advanced or metastatic disease and CPS ≥ 1 to pembrolizumab plus Lenvatinib or pembrolizumab alone (NCT04199104).

## Programmed Death-1 Inhibitor Plus Inducible Co-Stimulator of T Cells Agonist

The inducible co-stimulator of T cells (ICOS, or CD278) and its ligand (ICOSL) play important roles in memory and CTLs development and specific immune responses (189). ICOS and its pathway potentiates immunosuppression mediated by Tregs, but also induces antitumor responses when activated in CTLs (190, 191). Data on combination of anti-PD-1 with ICOS agonists are scarce, but synergy has been observed (192, 193). These data led to the rapid launching of a randomized phase 2 study evaluating the combination of ICOS agonists and anti-PD-1. Treatment naïve patients with advanced/metastatic HNSCC expressing PD-L1 (CPS ≥ 1) are randomized to receive pembrolizumab with GSK3359609 or placebo in the INDUCE-3 trial (NCT04128696). A second study will encompass HNSCC with or without PD-L1 expression. In this study, patients will be randomized to platinum-fluorouracil-pembrolizumab plus GSK3359609 or placebo (NCT04428333).

In addition to the aforementioned phase 3 studies, multiple phase 1/2 clinical trials with PD-1/PD-L1 inhibitors combined with a second drug targeting a variety of pathways are ongoing or have been completed (**Table 1**) (170–187). Studies for which data have been reported are summarized in **Table 1**. These clinical trials include patients that are immunotherapy-naïve (thus potentially addressing *de novo* resistance) and/or patients who have developed acquired resistance to anti-PD-1. While it is premature to elect a dominant combination strategy that will move forward to become a new standard of care, preliminary results for many of these studies are encouraging. Nonetheless, it is expected that resistance mechanisms will not be uniform in all patients, and biomarker-informed approaches will likely be needed to maximize the chances of achieving long-term successful outcomes, thus leading, in the future, to the development of precision immunotherapy for recurrent/metastatic HNSCCs, and ultimately earlier stage disease as well.

## Author Contributions

All authors contributed to the article and approved the submitted version.

## Conflict of Interest

LS is a paid advisor for Merck and reports receiving speaker’s bureau honoraria from Bristol-Myers-Squib and Merck. WW is a paid advisor for Pfizer, AstraZeneca, Merck, Bristol-Myers Squibb, and Roche/Genentech and reports receiving speaker’s bureau honoraria from AstraZeneca, Boehringer-Ingelheim, Roche/Genentech, Bristol-Myers Squibb, and Merck.

The remaining author declares that the research was conducted in the absence of any commercial or financial relationships that could be construed as a potential conflict of interest.
